# UWB-Based Self-Localization Strategies: A Novel ICP-Based Method and a Comparative Assessment for Noisy-Ranges-Prone Environments

**DOI:** 10.3390/s20195613

**Published:** 2020-10-01

**Authors:** Francisco Bonnin-Pascual, Alberto Ortiz

**Affiliations:** Department of Mathematics and Computer Science, University of the Balearic Islands, 07122 Palma de Mallorca, Spain; xisco.bonnin@uib.es

**Keywords:** UWB positioning system, point-to-sphere ICP, range filtering, ferromagnetic interference

## Abstract

Ultra-Wide-Band (UWB) positioning systems are now a real option to estimate the position of generic agents (e.g., robots) within indoor/GPS-denied environments. However, these environments can comprise metallic structures or other elements which can negatively affect the signal transmission and hence the accuracy of UWB-based position estimations. Regarding this fact, this paper proposes a novel method based on point-to-sphere ICP (Iterative Closest Point) to determine the 3D position of a UWB tag. In order to improve the results in noise-prone environments, our method first selects the anchors’ subset which provides the position estimate with least uncertainty (i.e., largest agreement) in our approach. Furthermore, we propose a previous stage to filter the anchor-tag distances used as input of the ICP stage. We also consider the addition of a final step based on non-linear Kalman Filtering to improve the position estimates. Performance results for several configurations of our approach are reported in the experimental results section, including a comparison with the performance of other position-estimation algorithms based on trilateration. The experimental evaluation under laboratory conditions and inside the cargo hold of a vessel (i.e., a noise-prone scenario) proves the good performance of the ICP-based algorithm, as well as the effects induced by the prior and posterior filtering stages.

## 1. Introduction

Position estimation in GPS-denied environments is of great interest in a large variety of applications, including indoor mobile robotics. Generally speaking, the so-called Indoor Positioning Systems (IPSs)—that is, systems that continuously and in real-time determine the position of an object in an indoor environment—can be applied in these cases [1]. From the technological point of view, IPSs comprise Radio Frequency Identification (RFID)-, Infrared (IR)-, Ultrasound (US)-, ZigBee-, Wireless Local Area Network (WLAN)-, and Ultra-Wide-Band (UWB)-based approaches, to name but a few. It is well-known that each of these technologies has its own pros and cons. By way of example, RFID localization systems do not require Line-of-Sight (LOS) to operate, which is critical for IR-based devices, but the coverage of the former is smaller in comparison with other technologies; IR and US signals do not penetrate solid walls, while ZigBee and WLAN signals do; ZigBee, however, is vulnerable to a wide range of signal types using the same frequency; while the performance of WLAN-based systems can be affected by changes in the strength map of the operating area. Likewise, UWB systems allow high-accuracy positioning, but can be affected by the presence of metallic materials. These are only a selection of the considerations to be made; the reader is referred to [2] for a more detailed overview and a comparison of IPSs.

In this paper, we focus on UWB positioning systems, with the goal of estimating the position of one or more devices, generally named *tags*, which are moving through an environment where a set of devices/beacons named *anchors* have previously been placed. UWB-based IPSs typically measure the distance from the tag to each of the anchors (e.g., four for 3D pose estimation), and combine them to obtain the position estimate. Unfortunately, the quality of the measured ranges may be affected by noise, which consequently propagates through the calculations and affects the reliability of the position estimates [3]. Indeed, some manufacturers warn about unpredictable effects on range measurements because of the presence of metallic materials in the surroundings of the operation area, and hence they recommend ensuring a minimum distance (above 20 cm) between the anchors’ antennas and any metallic element (see, e.g., www.pozyx.io/technology/where-to-place-the-anchors).

In this work, we propose a new method that has exhibited good performance in these noise-prone environments. The main novelty of this method is that it makes use of the well-known Iterative Closest Point (ICP) algorithm to estimate the position of the tag. Toward this end, we modified ICP, which is typically used to find the translation and rotation between two point clouds, to compute the position of the UWB tag through the computation of point-to-sphere correspondences. To the best of our knowledge, this new method is the first ICP-like algorithm that produces position estimates from data provided by a UWB-based localization system.

For performance evaluation purposes, we compared the aforementioned ICP-based method with other UWB-based position estimation approaches based on trilateration, focusing on the assessment of their noise-tolerance capabilities. Moreover, we also evaluated the effect of attaching pre- and post-processing filters to each of the methods involved in the comparison: on the one side, the pre-processing stage filters the tag-to-anchor range measurements on an anchor-by-anchor basis, while on the other side, the post-processing stage filters the raw position estimates resulting from the calculations. Finally, the algorithm’s performance is assessed under laboratory conditions and—as already mentioned—within a particularly noise-prone environment, such as a cargo hold of a large-tonnage vessel. Once more, to the best of our knowledge, this is the first time that a UWB-based IPS has been evaluated inside a ship, and the obtained results are reported, which can be regarded as a secondary contribution.

The rest of the paper is organized as follows: Section 2 reviews main approaches in UWB-based position estimation methods; next, Section 3 overviews our methodology regarding UWB-based position estimation and details the pre- and post-processing stages; Section 4 describes the novel ICP-based method to estimate the UWB tag position; Section 5 overviews the different methods chosen for the comparative assessment, which is actually performed in Section 6, where we evaluate the performance of all the configurations considered, both under laboratory conditions and within a real, noise-prone environment; finally, Section 7 draws some conclusions about the new method, as well as about the experimental results reported.

## 2. UWB-Based Position Estimation

Among the different currently available possibilities, UWB technology has emerged as one of the leading core technologies for IPS development thanks to (1) the resilience of UWB ultra-short pulses to frequency-dependent absorption, (2) a relatively low cost and easy deployment, and (3) the ultimate accuracy which can be achieved. It is well known that one of the key points is the measurement of distances between the tag(s) and the anchors. In this regard, a rough classification of UWB-based position estimation methods can be stated according to the base estimation technique that is adopted [4]:

- **Time of Arrival (TOA)**. Algorithms in this category estimate the position of the tag computing the intersection between the circumferences (or spheres in 3D) centred at each anchor, whose radius is the estimated distance from the tag to the corresponding anchor. A survey reviewing several TOA methods can be found in [5]. In [6], the authors evaluate different TOA-based algorithms in a realistic indoor environment. As a real application example, a UWB system based on TOA is used in [7] for personnel localization inside a coal mine.
- **Time Difference of Arrival (TDOA)**.This category comprises algorithms which estimate the position of the tag considering the difference between the reception times in each anchor given a signal sent by the tag. These methods require some synchronization mechanism between the different devices, as well as significant bandwidth in comparison with other methods. In [8], the authors propose a TDOA method to operate in complex environments, specially under non-line-of-sight (NLOS) conditions. This method makes use of an Extended Kalman Filter (EKF) as a post-processing stage. Another practical example is [9], which describes a real-time positioning system intended for disaster aid missions.
- **Angle of Arrival (AOA)**.Methods in this category estimate the position of the tag using the direction of propagation of the signals sent by multiple sources (i.e., the anchors). The location is found from the intersection of the angle line for each signal source. The algorithms based on AOA have a higher complexity and their accuracy may decrease when the distance increases. Among the large number of AOA-based approaches that can be found in the literature, we can mention [10], which makes use of a KF and relies on a linear quadratic frequency domain invariant beamforming strategy, and [11], which presents a cooperative positioning method that makes use of all the sensor nodes instead of using only the anchors.
- **Received Signal Strength (RSS)**.These methods employ the signal strength as an estimator of the distance. Among the many RSS-based algorithms, we can differentiate two main strategies. On the one hand, approaches based on trilateration, where the distance estimates are used to guess the position of the tag using the same methods employed by TOA methods (see for example [12,13]). On the other hand, a strategy based on RSS fingerprinting, where a dataset needs to be generated during a previous learning stage for collecting RSS data throughout the environment. This dataset is later used to compare with the RSS online measurements to estimate the location (see for example [14]).
- **Hybrid algorithms**.Hybrid techniques aim is to increase the precision of the position estimates by means of the combination of two or more of the aforementioned strategies. These methods are typically more complex and of higher and more intensive computational cost. By way of example, [15] reports on an EKF based on a TDOA/RSS algorithm to localize a UWB tag inside underground mines under NLOS conditions, while [16] evaluates several TDOA algorithms and concludes that a combination of them improves the accuracy of position estimates.

For a complete survey of UWB-based positioning algorithms, the reader is referred to [4,17,18].

## 3. General Overview and Methodology

The point of departure of our method is the availability of a regularly updated set of anchor-tag ranges, so that any beacon-based positioning system able to supply these data is susceptible to adopt our method for position estimation. This requirement is usually satisfied by UWB-based IPS vendors (see, e.g., Pozyx (www.pozyx.io) and Decawave (www.decawave.com) TOA-based solutions).

Regarding the position estimation procedure itself, we organize it as a process involving the following tasks (which are not sequenced in this order):

(a) Estimation of the position of the tag given a set of ranges to the anchors;
(b) Selection of the best subset of anchors to obtain the most accurate position estimation for the estimation method;
(c) Pre-filter (denoise) the available ranges; and
(d) Post-process (filter) the estimated positions.

Our aim in this work is to address and assess the four blocks. Regarding block (a), in this paper, we propose a novel method based on a particularization of the ICP algorithm. To properly feed this block, which is detailed in Section 4, we select first the most suitable collection of anchors presumably leading to the best position estimate, addressing thus block (b) of the previous list.

Regarding (c), the idea behind introducing a previous stage is to improve the data used as input by the position estimation block [19]. As mentioned before, the estimated distances to the anchors can be affected by external disturbances because of the presence of metallic elements in the environment. For this reason, we consider the addition of a pre-processing stage to filter the distances to the anchors, so that only good distance estimates are used within the position estimation block. The rationale behind this is to prevent the position estimation process to provide results when the distances to the anchors are not of sufficient quality.

The pre-filtering process that we adopt comprises two stages: A peak filter (PF) and a moving average filter(MAF) [20,21]. On the one hand, the peak filter removes all those values whose absolute difference with the previous value is above a certain threshold. These values are considered as peaks and are discarded, while the surviving values are considered as valid measurements.

On the other hand, the moving average filter supplies smoothed distance estimates by computing the mean of the *N* last consecutive valid ranges received. Since the ranges are required to be consecutive, when a peak is detected, the moving average filter does not provide output values until *N* new consecutive valid measures are available again.

Finally, as for (d), we adopt a post-processing stage consisting in a Kalman Filter-based strategy for position estimates [22]. This block implements an EKF which combines position estimates with motion data supplied by an Inertial Measurement Unit (IMU). More precisely, we make use of the IMU orientation and linear accelerations.

As for the experimental methodology, in the experimental results section, we will consider different configurations for the previous organization: without pre- and post- filters, with only one of them, or with both, and at the same time using different position estimation blocks (a), being one of them the new ICP-based method described in Section 4 and being the others each one of the different estimation strategies based on trilateration which are reviewed in Section 5.

## 4. Point-to-Sphere ICP for UWB-Based Position Estimation

In this section, we propose a novel method for estimating the position of a UWB tag by means of a modified version of the well-known ICP algorithm. To the best of the authors’ knowledge, the ICP algorithm, which is widely used for computing the roto-translation between two point clouds (e.g., provided by a LiDAR or from a depth camera), has never been used with data provided by a UWB positioning system. Nevertheless, the method described in this section can also be used with data provided by other systems based on the distances measured from a moving device to a set of beacons situated at known locations.

Our method modifies the ICP standard algorithm by computing a point-to-sphere correspondence between the 3D position of each anchor and the sphere defined by the distance to the anchor (i.e., the sphere radius) and the previous known location of the tag (i.e., the sphere center). More formally, let $A=\{a_{1},\ldots ,a_{N}\}$ be the collection of N≥4 anchors located at known positions $\left\{l_{1},\ldots ,l_{N}\right\}$, at let us consider a moving tag situated at distances $\left\{r_{1},\ldots ,r_{N}\right\}$ from the anchors. We next consider the set of spheres $S=\{s_{1},\ldots ,s_{N}\}$ with radius $\left\{r_{1},\ldots ,r_{N}\right\}$ centred at the last known location of the tag *t*. Then, the point-to-sphere ICP algorithm estimates the 3D translation of the spheres (and therefore the tag) necessary to allow for the anchors $a_{i}\in A$ to lie on the surface of the corresponding spheres $s_{i}\in S$.

The point-to-sphere ICP algorithm proceeds similarly to the point-to-line version of ICP [23]. At each iteration *j*, the algorithm computes, for each anchor $a_{i}\in A$, the point $c_{ij}$ in the surface of the corresponding sphere $s_{i}\in S$ which is closer to the anchor location $l_{i}$. Being $\left(t_{x},t_{y},t_{z}\right)$ the coordinates of the last known position of the tag, and $\left(l_{ix},l_{iy},l_{iz}\right)$ the coordinates of the anchor $a_{i}$, the coordinates of the point $c_{ij}$ can be computed as:   

(1) $$\begin{array}{cc} c_{ij\;,\;x} & =t_{x}+r_{i}\cos\alpha \cos\beta \;,\\ c_{ij\;,\;y} & =t_{y}+r_{i}\sin\alpha \cos\beta \;,\\ c_{ij\;,\;z} & =t_{z}+r_{i}\sin\beta \;, \end{array}$$

where

(2) $$\begin{array}{cc} \alpha & =\tan^{-1}\frac{l_{iy}-t_{y}}{l_{ix}-t_{x}}\;,\\ \beta & =\tan^{-1}\frac{l_{iz}-t_{z}}{\sqrt{{\left(l_{ix}-t_{x}\right)}^{2}+{\left(l_{iy}-t_{y}\right)}^{2}}}\;. \end{array}$$

**Figure 1:**
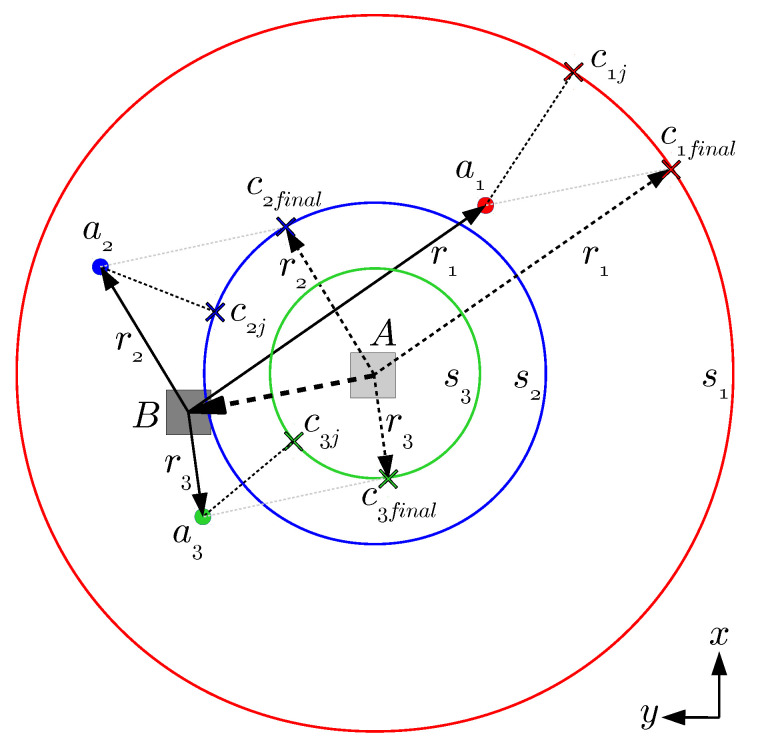
Example of point-to-circumference correspondences (2D case of point-to-sphere Iterative Closest Point (ICP)). The tag moves from point *A* to point *B*, while the anchors $a_{1}$, $a_{2}$ and $a_{3}$ are static. $c_{1j}$, $c_{2j}$ and $c_{3j}$ are the points over the three circumferences which are closer to the corresponding anchor in the current iteration *j*. $c_{1,\mathrm{final}}$, $c_{2,\mathrm{final}}$ and $c_{3,\mathrm{final}}$ are the final correspondences after ICP convergence.

Once all the correspondences $c_{ij}$ have been obtained for all anchors i∈{1,2,⋯,N}, we define the set of points $C_{j}=\left\{c_{1j},\ldots ,c_{Nj}\right\}$ for the current iteration *j*. This set of points $C_{j}$ is used next to estimate the translation of the tag by means of least squares point-to-point distance minimization, by which the optimum translation can be proved to be the average of distances between the anchors $l_{i}$ and the respective closest points $c_{ij}$. In the following iteration, the algorithm computes a new set of points $C_{j+1}$, which is then used to update the translation estimate (notice that this algorithm only computes a translation, while the point-to-line ICP algorithm computes a full roto-translation). To make all this easier to understand, Figure 1 illustrates graphically the point-to-sphere correspondence process by means of the 2D version (i.e., the point-to-circumference correspondence process).

The ICP loop iterates until the update in the estimated translation is below a certain threshold, that is, convergence is achieved, or a maximum number of iterations is reached. Considering the typically reduced number of anchors used in UWB positioning, together with the fact that ICP can start from the previous estimate, the point-to-sphere ICP algorithm usually converges in a few iterations—around 50, and typically less than 200 irrespective of the starting position employed (e.g., $t_{0}=\left(0,0,0\right)$). A description in pseudo-code of the point-to-sphere ICP algorithm can be found in Algorithm 1.
**Algorithm 1** Point-to-sphere ICP algorithm to estimate the position of the UWB tag1:**procedure**pointToSphereICP($L,R,t,\delta_{min},max\_iter$)2:    $L=\{l_{1},\ldots ,l_{N}\}$: anchors’ 3D positions3:    $R=\{r_{1},\ldots ,r_{N}\}$: distances from the tag to the anchors4:    *t*: starting estimate of the tag position, such as the last estimate or (0,0,0) the very first time5:    $\delta_{min}$: smallest position update to iterate once more6:    *max_iter*: maximum number of iterations to stop ICP7:    δ←∞, *num_iter*← 08:    **while** $(\delta >\delta_{min}$) **and** (*num_iter*< *max_iter*) **do**9:        C←**getClosestPoints**(L,R,t)                ▹ closest points obtained from Equation (1) and (2)10:        U←L−C                                                ▹ set of 3D translations required for each sphere11:        *mean_update* ←**average**(U)                        ▹ average update for each axis (from closed-form12:                                                                                ▹ solution of the underlying least-squares problem)13:        t←t+*mean_update*                                                                ▹ update the 3D position of the tag14:        δ←**norm**(*mean_update*)                                                                ▹ L2 norm of the update vector15:        *num_iter* ←*num_iter*+ 116:    **end while**17:    **return** *t*                                                                ▹ return the updated 3D position of the tag18:**end procedure**

Further, for higher robustness of point-to-sphere ICP, we enhance Algorithm 1 adopting a RANSAC-like estimation strategy [24]. That is to say, we consider random sets of m∈{4,⋯,N} anchors/ranges, apply Algorithm 1 to these minimum sets and determine the number of inliers among the full set of *N* available anchors/ranges. For inlier definition, we use the final point-to-sphere distance resulting for each anchor/range after ICP:

(3) $$d_{\text{point-to-sphere},\;i}={∥c_{i,\mathrm{final}}-l_{i}∥}_{2}$$

that is, an anchor/range $a_{i}/r_{i}$ is an inlier if $d_{\text{point-to-sphere},\;i}<\tau_{inl}$, for a given threshold $\tau_{inl}$. Finally, a set of anchors/ranges is considered the best set if it gives rise to the highest number of inliers, or, in case of tie, the sum of point-to-sphere distances is the lowest. Once the set of best anchors/ranges is available, we find the updated position applying Algorithm 1 to the corresponding set of inliers. Notice that, if the number of anchors is low, one can consider all possible combinations instead of a lower amount, as done by the original formulation of RANSAC.

To finish, it is worth mentioning that our method is also able to operate when, sporadically, less than four ranges are available because the remaining anchors are too distant, due to the presence of obstructing obstacles, because of punctual electromagnetic interference, etc. Under these conditions, Algorithm 1 can employ the available ranges to estimate the position of the tag, although at the expense of a higher error, which will depend on the number of available anchors and their locations. This makes it possible to operate in highly dynamic environments where other UWB positioning methods can not be used. However, although this is possible, we cancel the estimation process when not enough inliers can be found (at least *m*), and the method waits for the next set of ranges, in line with the idea of only supplying reliable position estimates.

**Figure 2:**
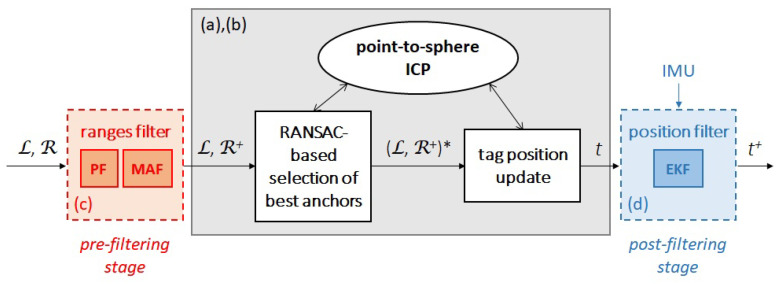
Block diagram of the full version of the ICP-based position estimation algorithm: L is the set of anchor locations, R is the set of ranges, $R^{+}$ is the set of filtered ranges, ${\left(L,R^{+}\right)}^{*}$ denotes the best set of anchors/ranges, *t* is the tag position, and $t^{+}$ is the filtered tag position. The dashed boxes used for the pre- and post-filtering stages denote that they can be removed. The gray box refers to the section that would be replaced by any of the methods overviewed in Section 5. (**a**–**d**) as defined in Section 3.

Figure 2 depicts graphically the ICP-based algorithm, including the pre- and post-filtering stages which would also be attached to the approaches described in Section 5.

## 5. Alternative Strategies

As alternative strategies to compare with point-to-sphere ICP, we consider three position estimation strategies based on trilateration. Trilateration can be described as a geometric method to find the location of a point based on the geometry of spheres, circles, or triangles. In the three-dimensional case, this method requires the location of at least three known points (e.g., the anchors) and the distances from all of them to the position to be determined (e.g., the UWB tag).

**Figure 3:**
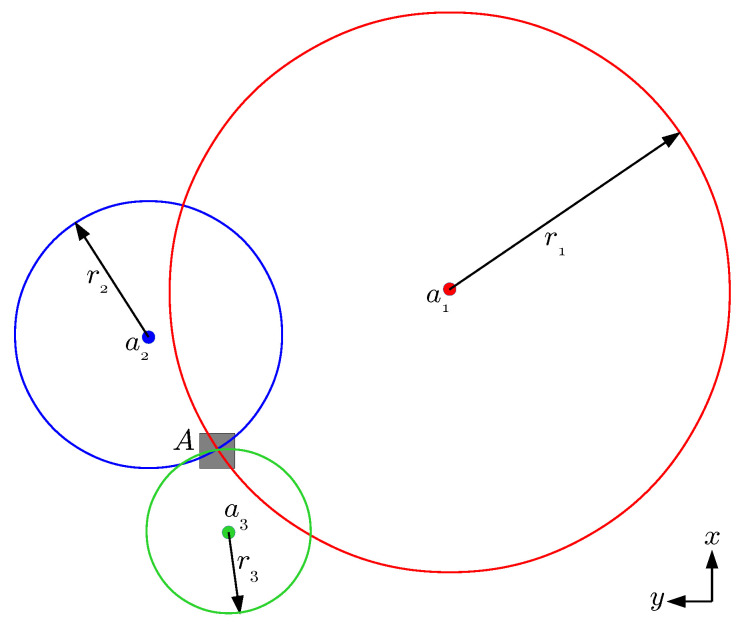
Trilateration example for the 2D case. The intersection of the three circumferences with radius $r_{1}$, $r_{2}$ and $r_{3}$, respectively centred at the anchors $\left(a_{1},l_{1}\right)$, $\left(a_{2},l_{2}\right)$ and $\left(a_{3},l_{3}\right)$, is used to compute the position of the tag situated at point *A*.

To solve for the position of the tag, the intersection of the spheres involved has to be found, using the distance between the tag and the corresponding anchor as the respective sphere radius. For a better understanding, see Figure 3, which shows this intersection for the 2D case. For the case of three anchors, the 3D position of the tag $t=(t_{x},t_{y},t_{z})$ can be computed from the equations of the three spheres:

(4) $$\begin{array}{c} {r_{1}}^{2}={\left(t_{x}-l_{1x}\right)}^{2}+{\left(t_{y}-l_{1y}\right)}^{2}+{\left(t_{z}-l_{1z}\right)}^{2},\\ {r_{2}}^{2}={\left(t_{x}-l_{2x}\right)}^{2}+{\left(t_{y}-l_{2y}\right)}^{2}+{\left(t_{z}-l_{2z}\right)}^{2},\\ {r_{3}}^{2}={\left(t_{x}-l_{3x}\right)}^{2}+{\left(t_{y}-l_{3y}\right)}^{2}+{\left(t_{z}-l_{3z}\right)}^{2}, \end{array}$$

where $l_{i}=\left(l_{ix},l_{iy},l_{iz}\right)$ is the location of anchor *i*, and $r_{i}$ is the distance between anchor *i* and the tag.

As already mentioned, for the comparative assessment with the point-to-sphere ICP, we consider three alternative strategies regarding the anchor selection. They all are detailed in the following sections.

### 5.1. RSS-Based Method

This method makes use of the RSS indicator to select the four anchors with highest values. Once the spheres are selected and ordered by this indicator, the method proceeds to compute the position of the tag from Equation (4) using the first three spheres, while the calculations for the fourth sphere are saved if they are not necessary. Three different situations may occur when the intersection between three spheres is considered:

(1) the three spheres intersect in a single point (ideal case),
(2) the circumference resulting from the intersection between the two first spheres does not intersect with the third sphere, and
(3) the circumference resulting from the intersection between the two first spheres intersects with the third sphere at two points.

**Figure 4:**
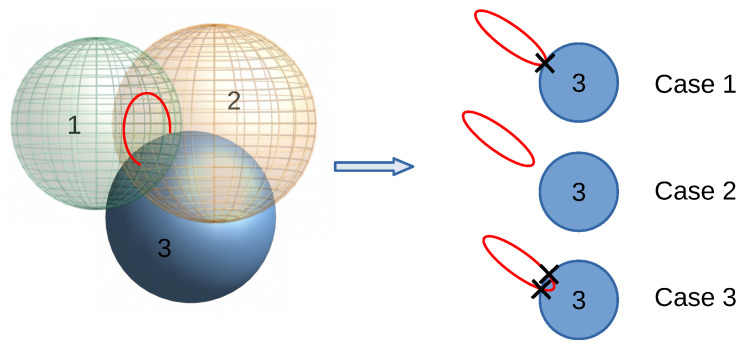
Three different cases for the intersection of three spheres.

These three cases are depicted in Figure 4. In the second case, the intersection between the three spheres is accepted when the distance between the circumference resulting from the intersection of the first two spheres and the third sphere is below a certain threshold. Otherwise, the algorithm does not provide solution for the given anchors and it waits for the next distance measurements. In the third case, the algorithm selects the intersection point which is closest to the surface of the fourth sphere. Hence, this algorithm requires at least four anchors/spheres to proceed.

To solve Equation (4) for the selected anchors, the anchors’ coordinates are transformed to an auxiliary coordinate frame centred at the location of the first anchor (i.e., the anchor with the highest RSS value) with the *x*-axis pointing to the second anchor, and so that the XY plane is defined with the third anchor. After this reference frame change, the first case (i.e., the three spheres intersecting in a single point) takes place when

(5) $${r_{1}}^{2}-x^{2}-y^{2}=0,$$

where (x,y) is the intersection point in the auxiliary coordinate frame. The second case occurs when

(6) $${r_{1}}^{2}-x^{2}-y^{2}<0,$$

and the third case takes place when

(7) $${r_{1}}^{2}-x^{2}-y^{2}>0.$$

Notice that the intersection point (if any) is always located in the XY plane of the auxiliary coordinate frame, and thus its *z* coordinate is always 0. The coordinates of the UWB tag in this reference system can next be obtained by means of:

(8) $$\begin{array}{cc} t_{x} & =\frac{{r_{1}}^{2}-{r_{2}}^{2}+{l_{2x}}^{2}}{2\;l_{2x}},\\ t_{y} & =\frac{{r_{1}}^{2}-{r_{3}}^{2}+{l_{3x}}^{2}+{l_{3y}}^{2}}{2\;l_{3y}}-\frac{l_{3x}\;t_{x}}{l_{3y}},\\ t_{z} & =\sqrt{{r_{1}}^{2}-{t_{x}}^{2}-{t_{y}}^{2}}, \end{array}$$

where $r_{1}$ to $r_{3}$ are the distances to the four anchors chosen and $l_{2}$, $l_{3}$ and $l_{4}$ are the positions of the second, third and fourth anchors in the auxiliary reference frame. Then, if $t_{z}\neq 0$, two situations may occur:

- ${r_{1}}^{2}-{t_{x}}^{2}-{t_{y}}^{2}<0$ (case 2 above), that is, there is no intersection between the spheres, and
- ${r_{1}}^{2}-{t_{x}}^{2}-{t_{y}}^{2}>0$ (case 3 above). In this case, we compute the Euclidean distance from the tag to the fourth anchor considering the positive and negative solutions for $t_{z}$, and we select the solution which leads to the shortest distance.

Finally, the estimated position of the tag must be transformed back to the original reference system used by the UWB device.

### 5.2. Minimum Discrepancy-Based Method

In this case, we apply the same steps as the RSS-based method to compute the position of the tag, although we select the four anchors in a different way. Indeed, this method tries all the combinations of four anchors, among all the available anchors, and selects the one which leads to the minimum trilateration discrepancy. Considering four specific anchors, the trilateration discrepancy is computed as the mean of the differences between the measured anchor-tag distances and the distances computed from the estimated tag position (estimated using these four anchors) to the position of each one of these anchors. In other words, the optimum subset B⊂A is such that |B|=4 and:

(9) $$B=\underset{B^{\prime }\subset A}{\arg\;\min}\sum_{i=1}^{N}\frac{\left|r_{i}-\left(\sqrt{{\left(t_{B^{\prime }x}-l_{ix}\right)}^{2}+{\left(t_{B^{\prime }y}-l_{iy}\right)}^{2}+{\left(t_{B^{\prime }z}-l_{iz}\right)}^{2}}\right)\right|}{N},$$

where $t_{Bx}$, $t_{By}$ and $t_{Bz}$ are the coordinates of the tag position estimated using the subset of anchors B and as described in Section 5.1.

### 5.3. Least Squares-Based Method

Unlike the previous methods, this method makes use of all the available anchors to estimate the position of the UWB tag. This is performed through a least squares formulation which can be explained starting from the following equations corresponding to the *N* spheres:

(10) $$\begin{array}{cc} {r_{1}}^{2}={\left(x-x_{1}\right)}^{2}+(y & -y_{1}{)}^{2}+{\left(z-z_{1}\right)}^{2},\\ \; & \vdots \\ {r_{N}}^{2}={\left(x-x_{N}\right)}^{2}+(y & -y_{N}{)}^{2}+{\left(z-z_{N}\right)}^{2}. \end{array}$$

The subtraction of the last equation from the preceding ones, gives rise to the N−1 following equations:

(11) $$\begin{array}{cc} 2\left(x_{N}-x_{1}\right)x+2\left(y_{N}-y_{1}\right)y+ & 2(z_{N}-z_{1})z=\\ {r_{1}}^{2}-{r_{N}}^{2}- & {x_{1}}^{2}-{y_{1}}^{2}-{z_{1}}^{2}+{x_{N}}^{2}+{y_{N}}^{2}+{z_{N}}^{2},\\ \; & \vdots \\ 2\left(x_{N}-x_{N-1}\right)x+2\left(y_{N}-y_{N-1}\right)y+ & 2(z_{N}-z_{N-1})z=\\ {r_{N-1}}^{2}-{r_{N}}^{2}- & {x_{N-1}}^{2}-{y_{N-1}}^{2}-{z_{N-1}}^{2}+{x_{N}}^{2}+{y_{N}}^{2}+{z_{N}}^{2}. \end{array}$$

Using matrix notation, we can express the previous system of equations as:

(12) Ap=b

where

$$A=\left[\begin{array}{ccc} 2(x_{N}-x_{1}) & 2(y_{N}-y_{1}) & 2(z_{N}-z_{1})\\ \vdots & \vdots & \vdots \\ 2(x_{N}-x_{N-1}) & 2(y_{N}-y_{N-1}) & 2(z_{N}-z_{N-1}) \end{array}\right],$$

$$p=\left[\begin{array}{c} x\\ y\\ z \end{array}\right]$$

and

$$b=\left[\begin{array}{c} {r_{1}}^{2}-{r_{N}}^{2}-{x_{1}}^{2}-{y_{1}}^{2}-{z_{1}}^{2}+{x_{N}}^{2}+{y_{N}}^{2}+{z_{N}}^{2}\\ \vdots \\ {r_{N-1}}^{2}-{r_{N}}^{2}-{x_{N-1}}^{2}-{y_{N-1}}^{2}-{z_{N-1}}^{2}+{x_{N}}^{2}+{y_{N}}^{2}+{z_{N}}^{2} \end{array}\right]$$

Finally, we obtain a standard least squares problem:

(13) $$\min_{p}\;{\left(Ap-b\right)}^{T}\left(Ap-b\right)$$

from which we can obtain a closed-form solution in terms of the pseudo-inverse of matrix *A*:

(14) $$p=A^{+}b={\left(A^{T}A\right)}^{-1}A^{T}b$$

Using this method, the solution *p* minimizes the root mean square error, what provides better results in case of inaccurate distance measurements. Similarly to the previous methods, in this case we also proceed with the calculations only when at least four anchors are available.

## 6. Comparative Evaluation

In this section, we report on the performance evaluation of the point-to-sphere ICP algorithm in comparison to the trilateration-based methods reviewed in Section 5. Besides, we evaluate the effect of introducing the pre- and post-processing stages explained in Section 3 for all position estimation approaches described.

For this evaluation, we have used the Pozyx Creator UWB kit (www.pozyx.io/products-and-services/creator), for a total of eight anchors, which have been placed on the walls surrounding the testing area. Further, two different environments have been considered: inside a laboratory and in a noise-prone environment. In the laboratory experiments, we employ $\delta_{min}=0.05$ (m) and *max_iter* = 200 (both from Algorithm 1), while $\delta_{min}=0.03$ (m) and *max_iter* = 300 for the noise-prone environment experiments. In all cases, an anchor/range is considered an inlier within RANSAC according to $\tau_{inl}=0.5$ (m).

These environments, and the experiments carried out, are detailed in the following sections.

### 6.1. Laboratory Experiments

The laboratory experiments have been carried out within a 10 m × 5 m × 5 m (L × W × H) volume inside the Aerial Robotics Lab, at the University of the Balearic Islands. The eight anchors have been placed on the walls and floor of the laboratory, at heights ranging from 0 to 4 m. This laboratory is equipped with a motion tracking system which is able to provide very accurate motion estimation, and thus can be used as ground truth data for the UWB tag position during the evaluation. For performance assessment purposes, we considered three different trajectories:

- Trajectory 1—a rectangular trajectory of 5 × 2 m, performed at a constant height;
- Trajectory 2—a figure-eight-like trajectory of 5 × 2 m, performed at a constant height; and
- Trajectory 3—a rectangular trajectory of 5 × 2 m changing the height of the tag, where the height was 2.5 m for the two longer transects and 1.5 m for the two shorter transects.

These datasets have been generated by following the intended trajectories and manually holding the UWB tag with motion tracking markers attached to it. For further insight, Table 1 reports on the amount of noise in the tag-anchor ranges as supplied by the UWB kit for the eight anchors and the three different motion paths followed during the laboratory experiments. Toward this end, we determined the discrepancy between the ranges measured by the anchors and the true ranges calculated by means of the available ground truth motion data. The table shows, on an anchor-by-anchor basis, the average discrepancy and the corresponding standard deviation (as statistical measures of the ranges’ noise) and the maximum discrepancy (to illustrate worst cases), all for each anchor independently in order to account for favourable/non-favourable anchor placement during the experiments. As can be observed, the average error was up to around 10 cm, while the worst errors reached several meters.

In the following sections, we make use of the notation described next to refer to the different methods and data:

- The trilateration methods are denoted as *T_RSS*, *T_MIN* and *T_LS*, for, respectively, the RSS-based method, the minimum discrepancy-based method and the least squares-based method;
- The point-to-sphere ICP-based method is referred to as *ICP*;
- The position estimates provided by the Pozyx kit itself are denoted as *POZYX*; and
- The ground truth data supplied by the motion tracking system is labelled as *GT*.

During the laboratory experiments, the position estimation methods are evaluated for three different configurations: (1) standalone configuration (i.e., without pre- and post- filtering), (2) adding the pre-filtering stage, and (3) incorporating both the pre-filtering and the post-filtering stages.

#### 6.1.1. Results Using the Standalone Configuration

**Figure 5:**
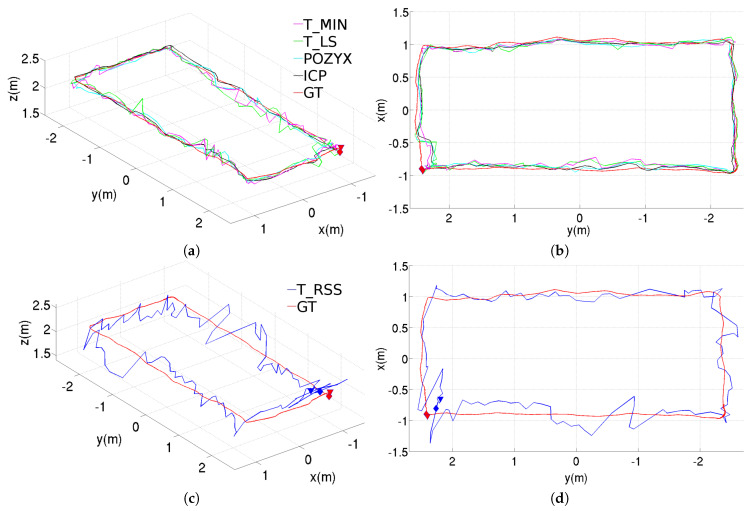
Position estimations provided by the different methods for the rectangular trajectory using the standalone configuration, results for the T_RSS method are shown separately to facilitate the comparison: (**a**) perspective and (**b**) top views for T_MIN, T_LS, POZYX and ICP, (**c**) perspective and (**d**) top views for T_RSS.

**Figure 6:**
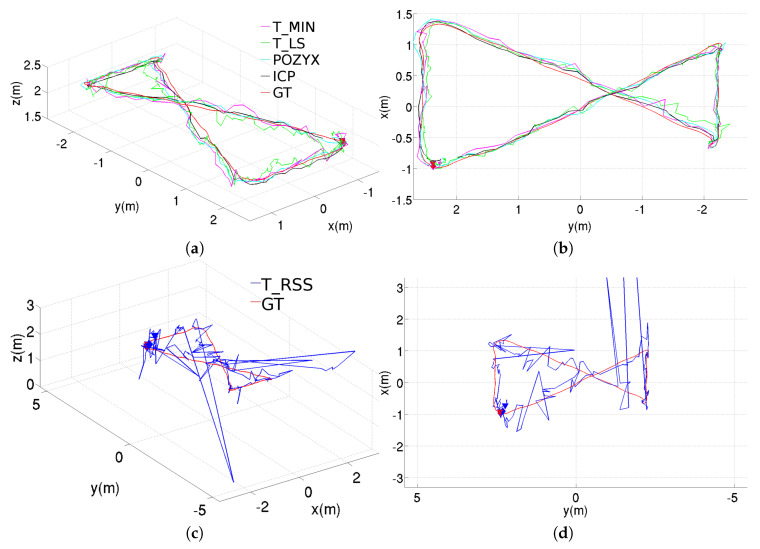
Position estimations provided by the different methods for the figure-eight-like trajectory using the standalone configuration, results for the T_RSS method are shown separately to facilitate the comparison: (**a**) perspective and (**b**) top views for T_MIN, T_LS, POZYX and ICP, (**c**) perspective and (**d**) top views for T_RSS.

**Figure 7:**
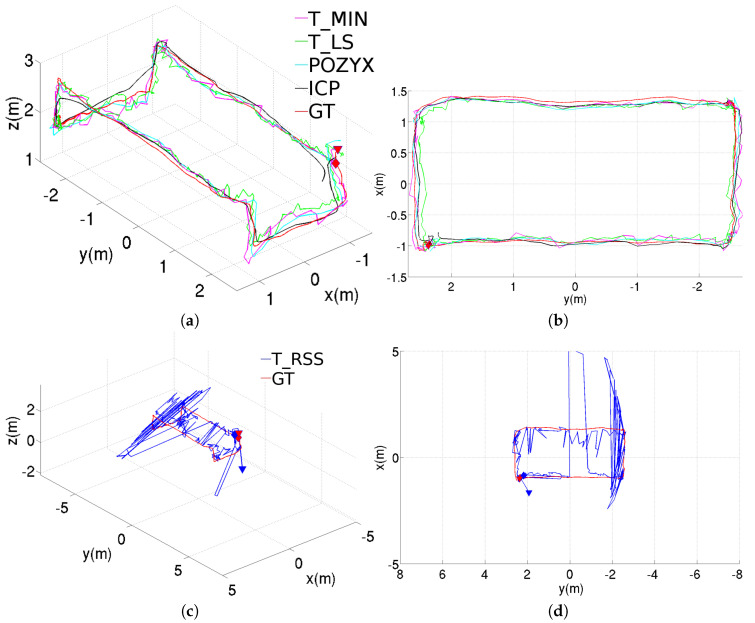
Position estimations provided by the different methods for the rectangular trajectory with changes in height using the standalone configuration, results for the T_RSS method are shown separately to facilitate the comparison: (**a**) perspective and (**b**) top views for T_MIN, T_LS, POZYX and ICP, (**c**) perspective and (**d**) top views for T_RSS.

Figure 5 shows the position estimation results obtained with the different methods, when these are used in standalone configuration. These results correspond to the rectangular trajectory, while the results provided through Figure 6 and Figure 7 correspond to, respectively, the figure-eight-like trajectory and the rectangular trajectory with changes in height. As can be seen in the three figures, in the laboratory, most of the methods present similar performances. Nevertheless, the T_RSS method leads to considerably noisier position estimates (in the figure, these are provided separately for a better visualization of the position estimates resulting from the rest of methods).

Table 2 quantitatively compares all methods for the rectangular trajectory inside the laboratory. The table reports on different metrics about the difference between position estimates and the GT data supplied by the motion tracking system, namely the mean, the standard deviation, the Root-Mean-Square Error (RMSE), the median, and the 90th, 95th, and 98th percentiles of the error [25]. Referring to the results obtained for the standalone configuration of each method, Table 2 shows that the performance of the ICP-based method is comparable to those of POZYX and T_MIN. It is worth noting that ICP leads to the lowest standard deviation and the lowest errors at 95th and 98th percentiles. Among the trilateration-based methods, T_MIN gives rise to the best results, followed by T_LS and, finally, T_RSS. This indicates that the selection of the anchors plays an important role: on the one hand, the subset of anchors which minimizes the trilateration error (used by T_MIN) seems to lead to better performance than considering all the anchors (as in T_LS), probably because the distance to some of the anchors is incorrectly estimated, possibly due to interferences from, for example, metallic elements in the walls and the floor. On the other hand, RSS does not seem to be the best indicator for selecting the anchors.

Similarly, Table 3 shows performance data for the figure-eight-like trajectory. When considering the standalone configurations, we can observe that the ICP-based method leads to the best values for all the metrics considered. Regarding the trilateration-based methods, the performance presented by these methods agree with what we have observed for the previous experiment, what reinforces our hypothesis about the importance of the anchors selection.

Finally, Table 4 reports on the third kind of trajectory, where the rectangular path is followed at different heights. Again, regarding the standalone configurations, the results of the ICP-based method are better than those for the three trilateration-based methods considered in this study.

#### 6.1.2. Results after Adding the Pre-Filtering Stage

**Figure 8:**
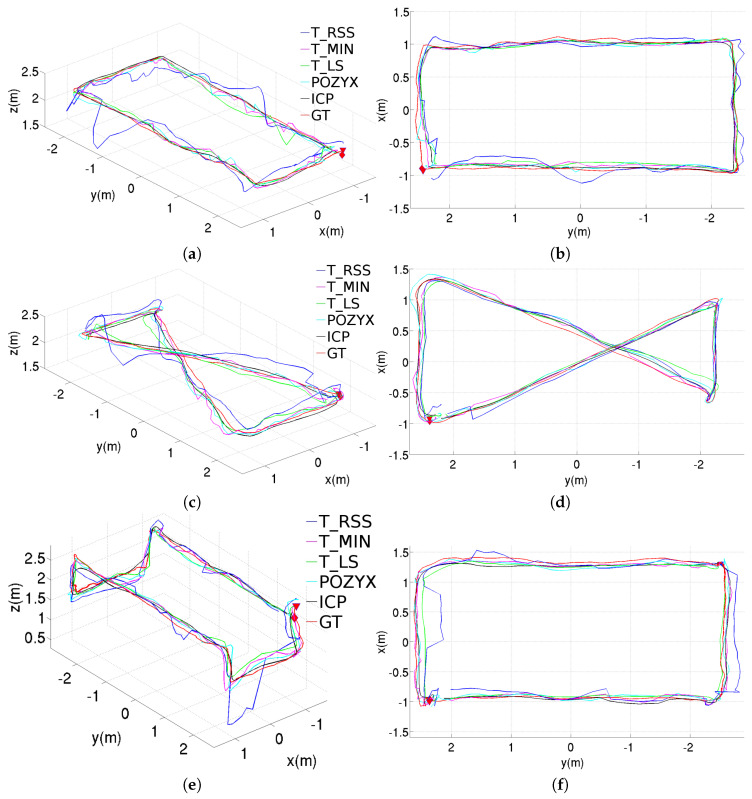
Position estimations provided by the different methods for the three trajectories performed inside the laboratory, results obtained using the pre-filtering stage: (**a**) perspective and (**b**) top views for the rectangular trajectory; (**c**) perspective and (**d**) top views for the figure-eight-like trajectory; (**e**) perspective and (**f**) top views for the rectangular trajectory with changes in height.

Figure 8 shows the estimated trajectories corresponding for the same three paths, but incorporating the pre-filtering stage to filter the anchor-tag distances. As can be observed, all the position estimates provided by the different methods now look smoother, being T_RSS the method which is more favored by the addition of this stage.

Regarding the numerical values of Table 2, Table 3 and Table 4, we can observe that the use of the pre-filtering stage leads, in general, to lower values of the different metrics for all the methods considered.

#### 6.1.3. Results after Adding the Pre- and Post-Filtering Stages

**Figure 9:**
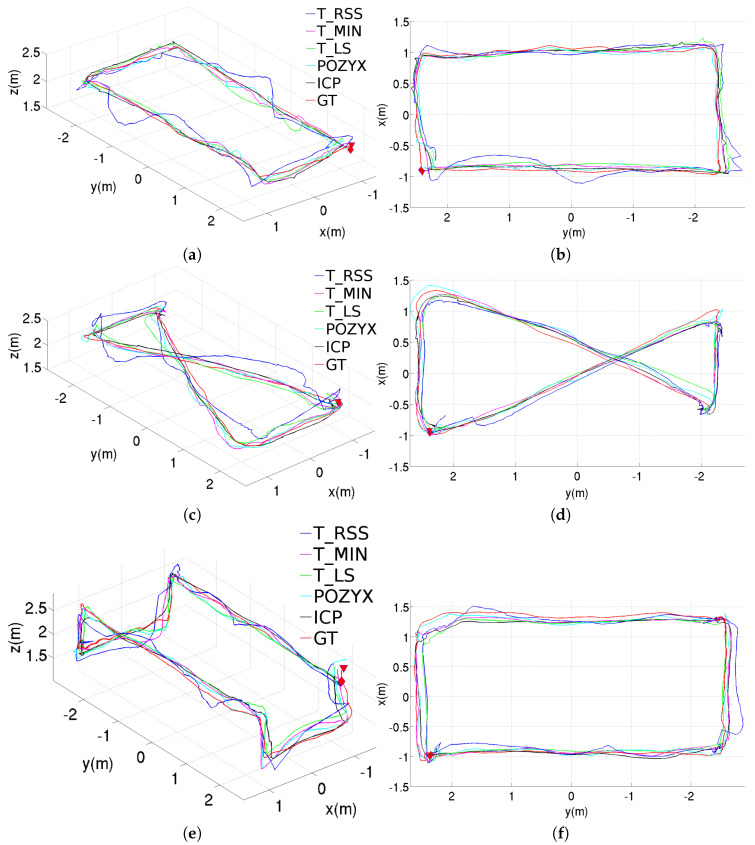
Position estimations provided by the different methods for the three trajectories performed inside the laboratory, results obtained using both the pre- and post-filtering stages: (**a**) perspective and (**b**) top views for the rectangular trajectory; (**c**) perspective and (**d**) top views for the figure-eight-like trajectory; (**e**) perspective and (**f**) top views for the rectangular trajectory with changes in height.

Figure 9 plots the estimated trajectories for the tree experiments carried out in the laboratory for the full configurations. In comparison with the trajectories plotted in Figure 8, the addition of the post-filtering stage leads to smoother trajectories, as expected.

Looking at the numerical performance data shown in Table 2, Table 3 and Table 4, one can observe that the results obtained after adding the EKF at the output of the pipeline are similar to the ones obtained using only the pre-filtering stage, or even slightly worse in some cases (probably due to the inherent delay introduced by this kind of filters). In any case, the performance of the post-filtering stage is expected to be more notorious in a non-UWB-favorable environment, where the incorporation of data from other sensors (e.g., an IMU) can really benefit position estimators with regard to using purely UWB-based methods.

### 6.2. Experiments in a Noise-Prone Environment

In this section, we report on some field experiments which have been carried out in one of the cargo holds of a Ro-Ro type vessel (typically intended for transporting cars, trucks, etc.). As a merchant ship, the cargo holds consist in metallic boxes, so that this kind of environment can be considered as a noise-prone scenario for a UWB positioning system.

**Figure 10:**
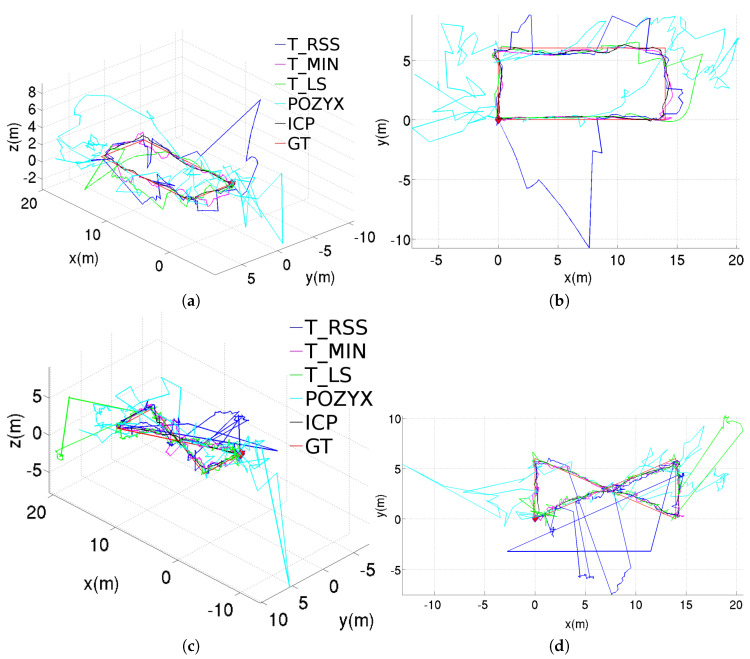
Position estimations provided by the different methods for the two trajectories performed inside the vessel hold, results obtained using both the pre- and post-filtering stages: (**a**) perspective and (**b**) top views for the rectangular trajectory; (**c**) perspective and (**d**) top views for the figure-eight-like trajectory.

Figure 10 plots the results obtained from the different UWB methods during a rectangular trajectory and a figure-eight-like trajectory, both performed inside one of the cargo holds of the aforementioned ship. All methods have been configured to make use of both the pre- and post-filtering stages. The performance exhibited in general for the other configurations (i.e., standalone and using only the pre-filtering stage) can be reported to be of low quality, in accordance to such noisy environment. In all plots, we make use of the trajectories labelled as GT in Figure 10 as reference trajectories for qualitative comparison, since, inside the cargo hold, there was no way to have access to accurate positioning data such as the ones provided by the motion tracking system of our laboratory. These reference trajectories were manually planned by means of a measuring tape and tracked during the experiments using reference lines painted on the floor. In the same way as for the laboratory experiments, the position estimates supplied by the manufacturer’s software are also shown in Figure 10 and labelled as POZYX.

As can be observed, the only methods which are able to adhere to the ground truth are T_MIN and ICP, being the latter the method which behaves better. The good performance of these methods is partially due to the good selection of the subset of anchors. The T_LS method is able to follow the trajectory most part of the time, but at certain points suffers from some large deviations due to the use of anchors whose range has been poorly estimated. As happened for the laboratory experiments, T_RSS gives rise to the worse results, which in this case cannot be sufficiently improved after incorporating the pre- and post-filtering stages. A special mention is made to the quality of the POZYX estimates, which are severely affected by the metallic environment, as already warned by the manufacturer.

## 7. Conclusions and Future Work

In this work, we have presented a novel method for UWB-based position estimation by means of point-to-sphere ICP. The method has been described and its performance has been compared with alternative position estimators based on trilateration. During the development of the proposed method, and the subsequent comparative evaluation, one of our concerns has been the quality of the anchor-tag distance estimations and thus to establish an adequate anchor selection process. Following with this, we have also considered as part of the performance evaluation the effect of incorporating a pre-processing stage that filters and improves the quality of the range estimates, which are in turn used as input for the position estimation method. Similarly, we have also evaluated the incorporation of a post-processing stage that filters the position estimates by means of non-linear Kalman filtering.

We have reported results for laboratory and field experiments, showing the good performance of the point-to-sphere ICP-based method, which outperforms the alternative position estimation methods considered in the paper. The results also allows us to confirm the importance of the anchors selection step: among the trilateration-based methods, T_MIN has led to the best performance in all experiments, since this method selects the subset of anchors which minimizes the trilateration error. A similar idea is implemented within the point-to-sphere ICP-based method, where RANSAC is used to choose the subset of anchors which provides the lowest global error.

The results of the experiments using the pre-filtering stage indicate that this step is useful to improve the range estimates that are subsequently used by all the methods evaluated. On the other side, the post-filtering stage based on an EKF has proved useful when the UWB devices are operating within a noisy environment, where data provided by other sensors can contribute to obtain more accurate position estimates.

Regarding the computational cost of the ICP-based approach, we have observed that, for the configurations we have considered, convergence is attained after a few iterations—around 50 if the previous estimate is used, and less than 200 irrespectively of the starting estimate—, what, in a standard computer, means execution times of the order of milliseconds, a time-lapse comparable to the computation time of the other methods involved in the comparison. Increasing the number of anchors will make the computational cost increase as well, although normally the bottleneck is rather on the time needed to collect the ranges from the different anchors instead of on the calculations.

Like any other UWB-based positioning method, the point-to-sphere ICP-based method can be affected by poor positioning of the anchors, what in turn can result in an ill-conditioned problem. Since our method is based on ICP, it may need some additional iterations to converge when the anchors are not properly situated. In this case, the update in the position estimate between iterations can be rather small, so that, depending on the stopping conditions used in the ICP loop, the algorithm might decide that convergence conditions are met and stop prematurely, giving rise to inaccuracies in the position estimates.

As for future work, we plan to improve the estimation of the tag’s height by tolerating better the lack of variation in the anchors heights, by means of the incorporation of additional sensors into the data fusion step. In particular, we are concerned with the use of the point-to-sphere ICP-based method for estimating the 3D position of a Micro-Aerial Vehicle (MAV) and, hence, the enhancements in height estimation can greatly contribute to improving the performance of the full system as a whole. The integration of the point-to-sphere ICP into a SLAM solution which is currently under development is another item which will be part of future–though relatively immediate–work.

## Figures and Tables

**Table 1:** Discrepancy between true tag-anchor ranges and measured ranges as supplied by the UWB kit involved in the laboratory experiments. All values are in meters.

Anchor	Trajectory 1			Trajectory 2			Trajectory 3		
	Mean	Std. Dev.	Max.	Mean	Std. Dev.	Max.	Mean	Std. Dev.	Max.
0	0.135	0.508	4.795	0.072	0.061	0.364	0.135	0.391	4.166
1	0.176	0.559	6.502	0.089	0.090	0.396	0.147	0.415	3.994
2	0.064	0.045	0.218	0.075	0.054	0.225	0.118	0.536	6.681
3	0.076	0.067	0.450	0.106	0.076	0.457	0.115	0.256	3.173
4	0.043	0.025	0.111	0.054	0.044	0.231	0.118	0.452	5.636
5	0.060	0.032	0.138	0.092	0.117	0.685	0.050	0.034	0.162
6	0.095	0.059	0.294	0.093	0.073	0.377	0.152	0.663	6.628
7	0.088	0.304	3.483	0.140	0.462	3.693	0.091	0.086	0.376

**Table 2:** Performance data for the rectangular trajectory. Values in red denote the three best values for each metric. All values are in meters.

Method	Configuration	Mean	Std. Dev.	RMSE	Median	90th per.	95th per.	98th per.
POZYX	—	0.113	0.053	0.125	0.105	0.203	0.213	0.221
	standalone	0.231	0.120	0.260	0.234	0.372	0.479	0.553
T_RSS	pre-filter	0.191	0.112	0.221	0.191	0.283	0.356	0.425
	pre- & post-filter	0.182	0.087	0.202	0.187	0.288	0.326	0.371
	standalone	0.117	0.056	0.129	0.116	0.179	0.207	0.249
T_MIN	pre-filter	0.118	0.052	0.129	0.119	0.190	0.206	0.229
	pre- & post-filter	0.119	0.048	0.128	0.120	0.183	0.203	0.231
	standalone	0.126	0.069	0.144	0.117	0.228	0.262	0.305
T_LS	pre-filter	0.124	0.048	0.133	0.118	0.193	0.235	0.243
	pre- & post-filter	0.132	0.049	0.141	0.131	0.199	0.246	0.249
	standalone	0.121	0.045	0.129	0.125	0.180	0.189	0.195
ICP	pre-filter	0.121	0.034	0.125	0.121	0.162	0.165	0.170
	pre- & post-filter	0.123	0.039	0.129	0.124	0.172	0.176	0.180

**Table 3:** Performance data for the figure-eight-like trajectory. Values in red denote the three best values for each metric. All values are in meters.

Method	Configuration	Mean	Std. Dev.	RMSE	Median	90th per.	95th per.	98th per.
POZYX	—	0.110	0.044	0.119	0.114	0.166	0.181	0.191
	standalone	0.501	0.830	0.969	0.281	0.827	1.239	4.012
T_RSS	pre-filter	0.236	0.100	0.256	0.234	0.371	0.389	0.464
	pre- & post-filter	0.239	0.112	0.264	0.240	0.395	0.418	0.442
	standalone	0.118	0.068	0.136	0.112	0.213	0.251	0.287
T_MIN	pre-filter	0.111	0.044	0.119	0.109	0.173	0.183	0.193
	pre- & post-filter	0.112	0.050	0.122	0.111	0.180	0.197	0.211
	standalone	0.125	0.076	0.147	0.111	0.235	0.255	0.315
T_LS	pre-filter	0.122	0.066	0.138	0.100	0.226	0.237	0.243
	pre- & post-filter	0.119	0.070	0.139	0.102	0.217	0.259	0.279
	standalone	0.081	0.043	0.092	0.075	0.139	0.162	0.189
ICP	pre-filter	0.078	0.046	0.090	0.066	0.142	0.176	0.181
	pre- & post-filter	0.090	0.050	0.103	0.091	0.165	0.194	0.200

**Table 4:** Performance data for the rectangular trajectory with changes in height. Values in red denote the three best values for each metric. All values are in meters.

Method	Configuration	Mean	Std. Dev.	RMSE	Median	90th per.	95th per.	98th per.
POZYX	—	0.103	0.060	0.119	0.100	0.180	0.211	0.250
	standalone	0.641	0.990	1.179	0.265	1.880	2.511	3.642
T_RSS	pre-filter	0.238	0.241	0.339	0.187	0.389	0.499	1.288
	pre- & post-filter	0.196	0.126	0.233	0.176	0.334	0.401	0.548
	standalone	0.120	0.080	0.145	0.102	0.212	0.269	0.344
T_MIN	pre-filter	0.116	0.081	0.142	0.102	0.216	0.258	0.354
	pre- & post-filter	0.129	0.083	0.153	0.112	0.247	0.300	0.327
	standalone	0.125	0.081	0.149	0.114	0.227	0.290	0.355
T_LS	pre-filter	0.116	0.079	0.141	0.105	0.208	0.251	0.352
	pre- & post-filter	0.133	0.084	0.157	0.118	0.259	0.299	0.342
	standalone	0.117	0.064	0.134	0.110	0.185	0.211	0.266
ICP	pre-filter	0.109	0.055	0.122	0.107	0.188	0.198	0.208
	pre- & post-filter	0.126	0.064	0.142	0.116	0.213	0.229	0.255
